# Robotic bilateral inguinal lymphadenectomy in penile cancer, development of a technique without robot repositioning: a case report

**DOI:** 10.3332/ecancer.2013.356

**Published:** 2013-09-26

**Authors:** Rene Sotelo, Marino Cabrera, Oswaldo Carmona, Robert de Andrade, Oscar Martin, Golena Fernandez

**Affiliations:** Instituto Médico La Floresta, Caracas 1060, Venezuela

**Keywords:** penile cancer, robotic surgery, lymphadenectomy, lymph nodes

## Abstract

**Introduction:**

Inguinal lymphadenectomy is the treatment of choice for patients with penile cancer and inguinal lymph node metastases. We describe the performance of the robotic bilateral inguinal lymphadenectomy technique without repositioning the robot in a patient with penile carcinoma and high risk for nodal metastases and no palpable lymph nodes.

**Materials and methods:**

A 64-year-old male patient was diagnosed with penile cancer (TNM: T3 N 0 M 0) and underwent a total penectomy with perineal urethrostomy. We performed a robotic bilateral inguinal lymphadenectomy four weeks after the penectomy.

**Results:**

The entire procedure was performed with the robot-assisted technique. The operative time, median estimated blood loss, and hospital stay was 360 min, 100 ml (50 ml in the right side and 150 ml in the left side), and three days, respectively. Metastatic nodes were present in both inguinal regions, with a yield of 19 lymph nodes on the right and 14 on the left. The patient presented with a left-side lymphocele that was drained at follow-up. No other complications were reported.

**Conclusion:**

Robotic bilateral inguinal lymphadenectomy secondary to penile cancer is feasible, safe, and provides a good performance. Prospective studies are required to include a larger number of patients and long-term monitoring to assess the results of this procedure in comparison with open and laparoscopic techniques.

## Introduction

The management of the inguinal region in patients with squamous penile cancer is very controversial. The main predictor of survival is lymph node involvement.

Lymphadenectomy is the treatment of choice for patients with inguinal lymph node metastases; it is considered as a standard management for palpable lymphadenopathy secondary to penile cancer and is recommended in the management of patients with clinically negative nodes at high risk of micrometastases [1].

The high risk of complications secondary to open lymphadenectomy led to the development of video/minimally invasive endoscopic techniques, which have the same indications to open surgery.

The robotic approach offers advantages such as increased magnification, ergonomic platform, and three-dimensional vision, allowing greater precision, dexterity, and a degree of freedom which is not achieved with standard laparoscopy.

We describe a technique of robotic bilateral inguinal lymphadenectomy without robot repositioning in a patient with pT3 penile carcinoma and no palpable lymph nodes but high risk of nodal metastases.

## Material and Methods

A 64-year-old male patient, who presented with bulky penile mass, pain, and secretion of 10 years of evolution, without improvement after homeopathic treatment, underwent a biopsy reporting moderately differentiated invasive squamous carcinoma. The medical history was negative for other medical conditions.

On physical examination, there was a 5-cm bulky mass occupying the middle third of the penis to the base with multiple ulcerations vegetating at coronal sulcus level. No palpable inguinal adenopathy was detected.

Extension studies such as abdominopelvic and chest CT reported no evidence of lymph node metastasis.

The patient underwent a total penectomy and perineal urethrostomy.

The histopathological study reported a 5 x 4-cm^2^ poorly differentiated and ulcerated squamous cell carcinoma, with pleomorphic cells with a mitotic index of 15 mitoses in ten fields of high magnification and perineural, corpora cavernosa, spongiosum, and proximal urethra with lymphatic infiltration. TNM: T3 N 0 M 0.

The patient was advised to undergo a robotic bilateral inguinal lymphadenectomy.

## Technique

Positioning the patient on a split-leg table or in low lithotomy allows bilateral dissection without repositioning the robot itself.

**Figure 1.**
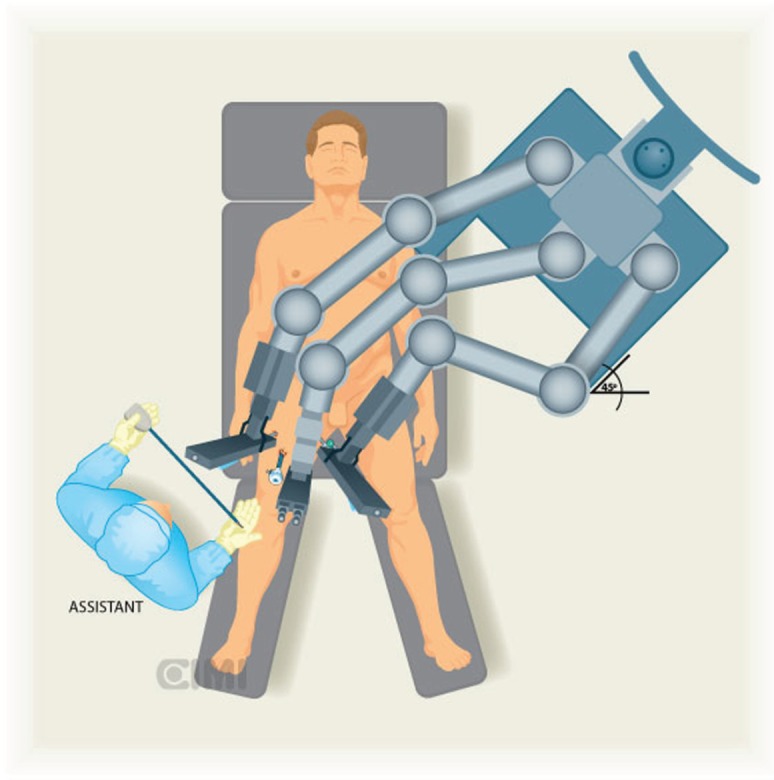
Assistant position for the right inguinal lymphadenectomy, the robot is located at 45° contralateral to procedure.

**Figure 2.**
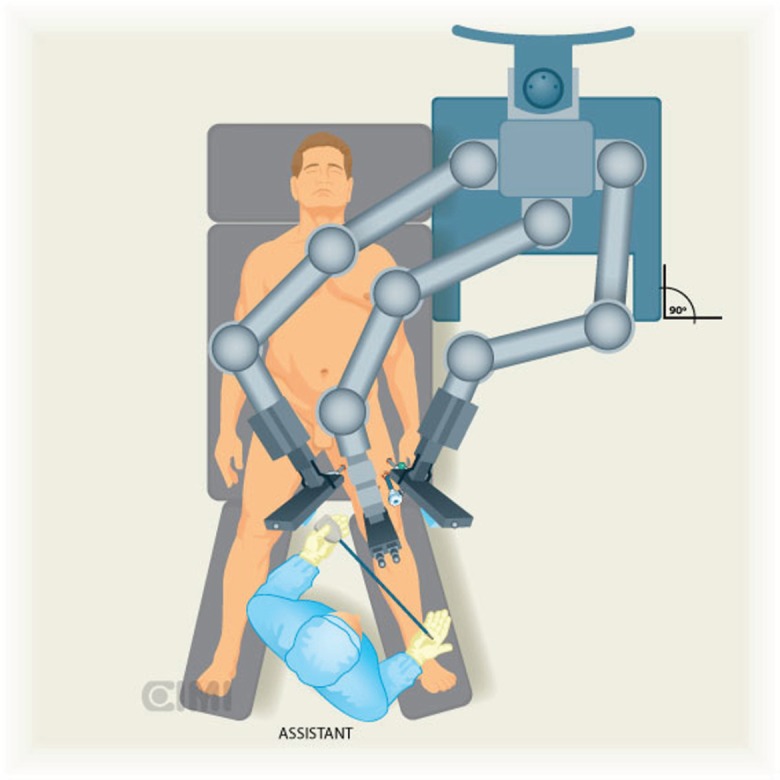
Assistant position for the left inguinal lymphadenectomy, the robot is located at 90° lateral to procedure.

A Foley catheter is inserted in sterile fashion after the inguinal and groin areas are prepared and draped. The assistant stands lateral to the right leg for right-side dissection, and between the legs for the left side (Figures 1 and 2).

**Figure 3.**
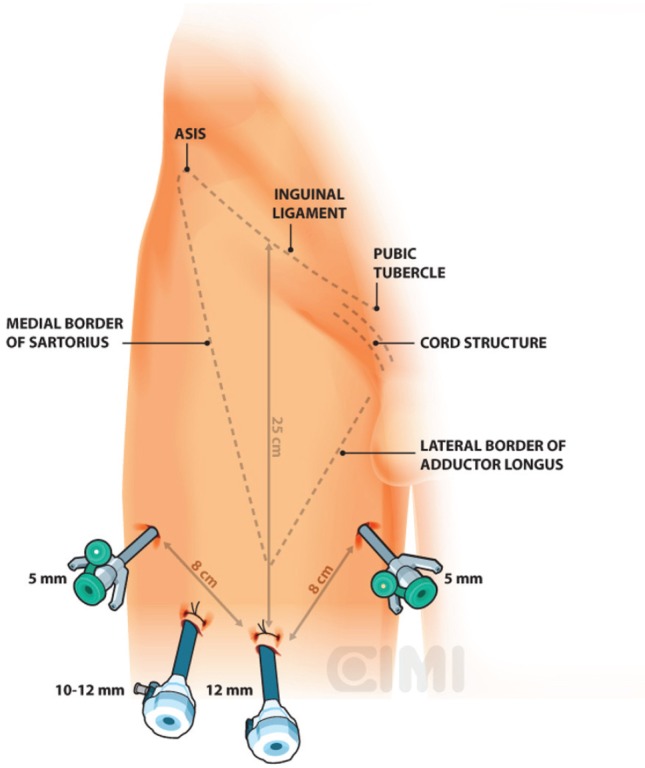
Right surgical procedure template. ASIS: anterior superior iliac spine.

**Figure 4.**
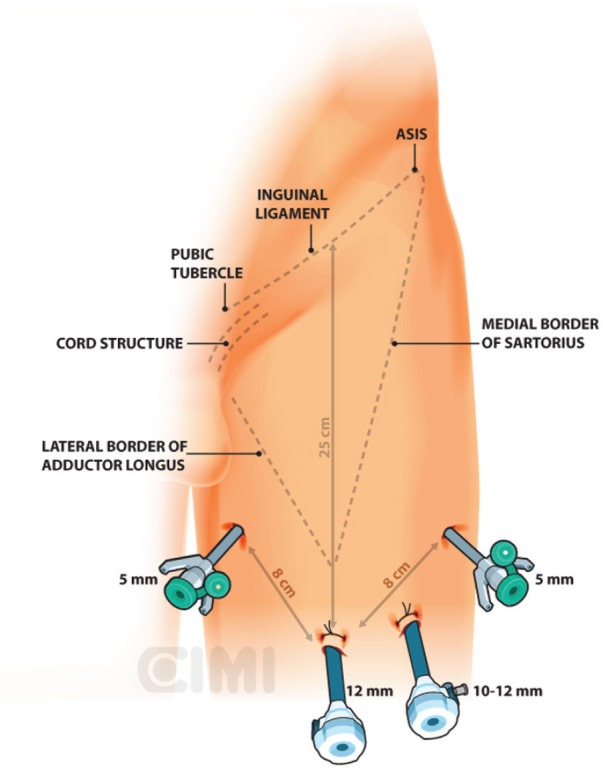
Left surgical procedure template.

Bony and soft tissue landmarks are marked on the skin surface, creating an inverted triangle, where the base is a line connecting the anterior superior iliac spine to the pubic tubercle, along the course of the inguinal ligament. The lateral boundary is the sartorius muscle angling towards the apex. The medial boundary is the adductor longus muscle, again extending towards the apex. These marks aid in correct trocar placement as well as delineating the extent of dissection (Figures 3 and 4).

The robot is located at 45° contralateral to the first procedure (right side) and lateral to the patient in the second procedure (left side).

**Figure 5.**
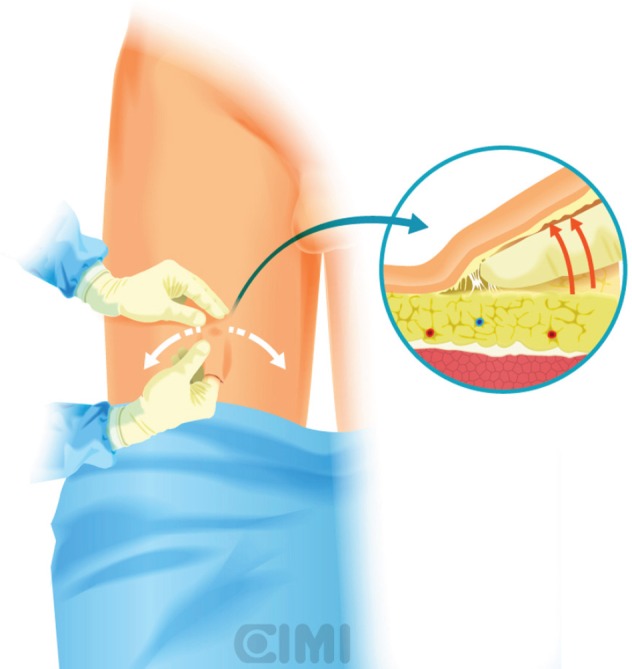
Guided finger subcutaneous creation of workspace.

**Figure 6.**
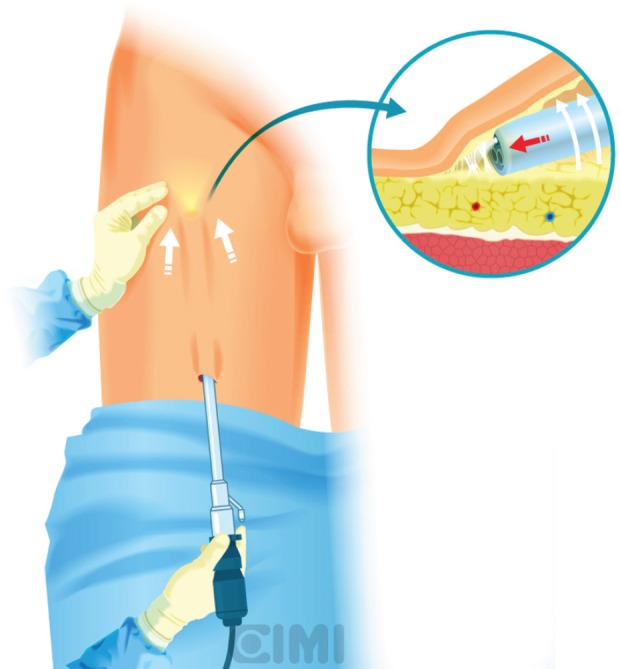
Endoscope subcutaneous workspace creation by sweeping with the lens itself.

For the right side, a 2-cm incision is made 3 cm below the inferior aspect of the femoral triangle, ~25 cm below the inguinal ligament. A white subcutaneous layer is identified, which corresponds to Scarpa’s fascia. Sweeping finger dissection is used to develop the potential space beneath Scarpa’s fascia to develop the skin flaps at the apex of the triangle out in both directions to two additional 8-mm ports. These two primary robotic 8-mm ports are placed with finger-guided techniques, laterally and medially as shown in Figures 5 and 6.

**Figure 7.**
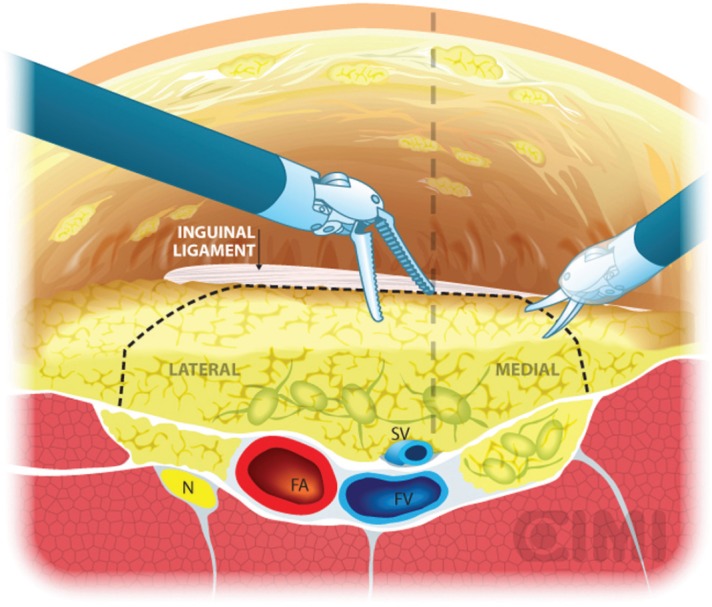
Subcutaneous surface robot vision under Scarpa’s fascia flap.

A subcutaneous workspace is extended with the endoscope by sweeping with the lens itself. The aim of this step is to create a superficial subcutaneous flap under Scarpa’s fascia (Figure 7). An alternative, after the initial finger dissection, is to use a 12-mm Origin balloon port trocar (Origin Medsystems Inc, Menlo Park, California, United States), set at 25-mm Hg for 10 min to create the space [9].

The workspace is expanded with CO_2_ insufflation at a pressure of 15 mmHg.

A 0° 10-mm lens is inserted, and one additional intervening 10-mm assistant port is placed between the camera and primary 8-mm working port on the assistant side.

The robot is positioned on the left side of the patient and docking to the right inguinal region, as shown in Figures 1 and 2.

**Figure 8.**
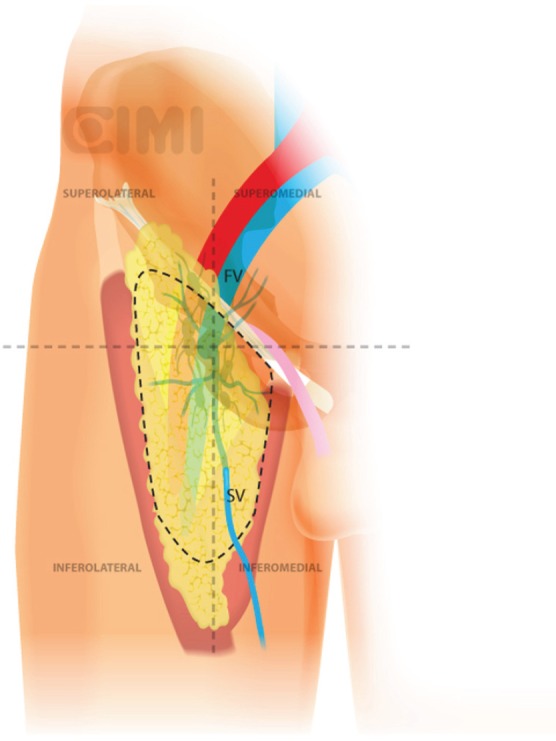
Dissection boundaries of inguinal lymphadenectomy. SV: saphenous vein; FV: femoral vein.

Our preferred instrument is bipolar Maryland or PK forceps in the left robotic arm and monopolar scissors in the right arm to dissect the membranous and lymphatic tissue just to the depth of the Camper’s fascia. Every effort is made to completely develop the anterior working space to the inguinal ligament. The inguinal ligament is usually identified at the end of this dissection as being a transverse structure with white fibres, marking the superior limit (Figure 8).

The boundaries of the dissection extend from the inguinal ligament superiorly, the sartorius muscle laterally, and the adductor longus muscle medially. One will be able to spare the saphenous vein in most of the cases, and the small branches of the femoral artery and vein may be clipped and divided (Figure 8).

Identification of the adductor longus and sartorius muscles is facilitated by identifying the fascia of the respective muscles and correlating to the previously made skin markings. The medial spermatic cord is seen medially. Inadvertent dissection to the depth of the fascia lata is apparent when reddish muscular fibres are seen.

With blunt dissection, the node packet can be rolled inwards on both sides. This manoeuvre is continued inferiorly as much as possible from both sides to define inferior apex of the nodal packet. The saphenous vein will be identified as it crosses the internal border of the dissection near the apex of the femoral triangle, and following it brings you to the saphenous arch until its junction with the superficial femoral vein at the fossa ovalis.

**Figure 9.**
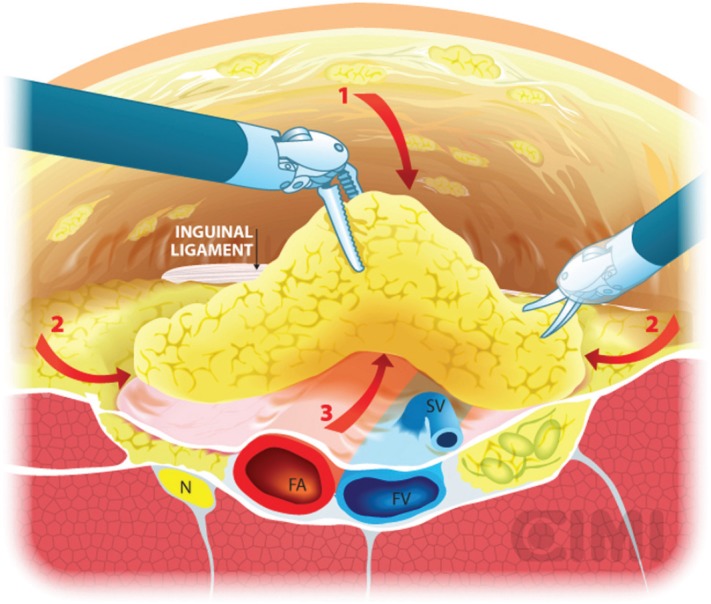
Steps for nodal dissection package, axial vision. SV: saphenous vein; FV: femoral vein; FA: femoral artery; N: nerve.

**Figure 10.**
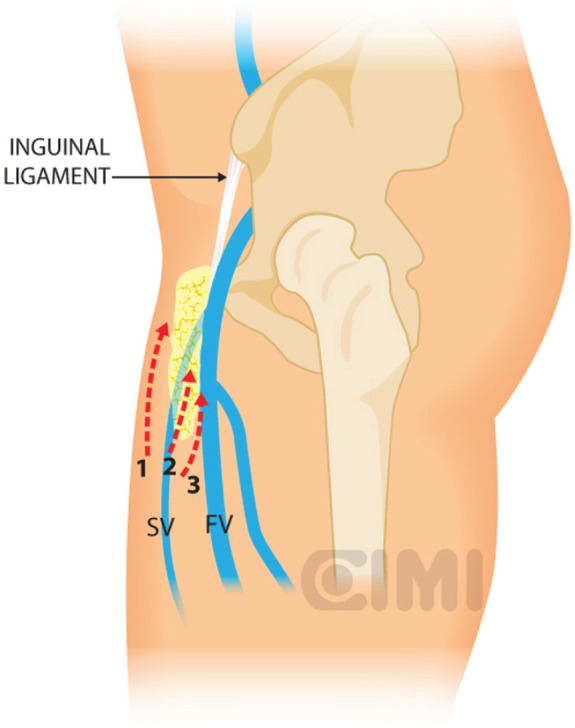
Steps for nodal dissection package, lateral vision. SV: saphenous vein; FV: femoral vein.

The dissection continues superiorly, where the packet is dissected off the fascia lata with a combination of sharp and blunt dissection. Typically, the nondominant hand lifts the packet and the monopolar scissors in the dominant hand advances the dissection. After encountering the fossa ovalis, the packet is dissected away at its superolateral and superomedial limits, thereby narrowing the packet and pulling it away from the inguinal ligament. At this point, the superficial and deep planes of dissection join and separate the package from the inguinal ligament (Figures 9 and 10).

With the nodal packet circumferentially dissected to the saphenous arch, where the tributaries veins are clipped. Characteristic pulsation of the femoral artery serves as a nearby landmark. If possible, the package will be released from the saphenous vein. If not, it can be ligated in the saphenous arch with weck clips or an endovascular stapler. One must always attempt to preserve the saphenous vein whenever possible to reduce the risk of postoperative lymphoedema [17].

The specimen is removed in an impermeable sac and extracted after extending the camera trocar incision. Frozen section results determine whether deep ipsilateral dissection will be required. We typically begin to create the working space in the other leg while waiting for the results. If the primary tumour is high grade with infiltration to corpora cavernosum or urethra, risk for deep node positivity is high enough that one should proceed without frozen section results.

**Figure 11.**
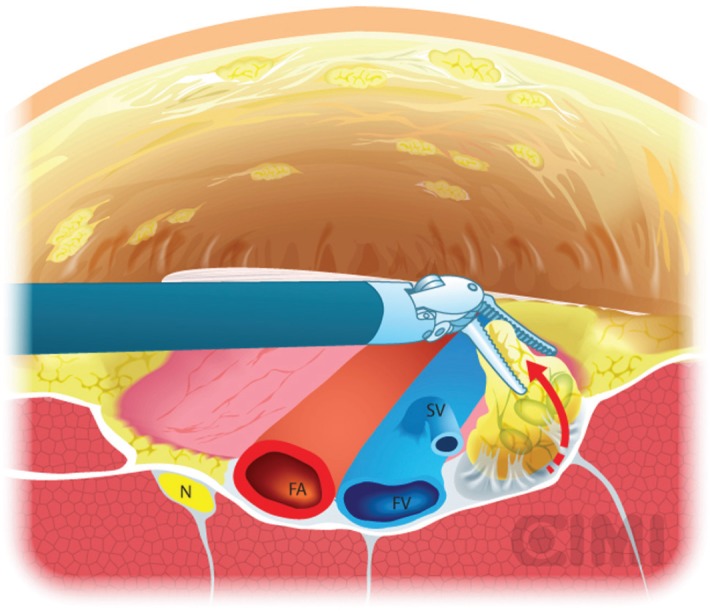
Deep pelvic node dissection. SV: saphenous vein; FV: femoral vein; FA: femoral artery; N: nerve.

For the deep pelvic node dissection, re-establish the pneumoperitoneum, open the fascia lata medial to the saphenous arch and expose the saphenofemoral junction. The inferomedial dissection around the femoral vein will enable resection of the deep inguinal nodes [9]. This should be continued to the level of the femoral canal and until the pectineus muscle is seen to insure complete nodal retrieval [18] (Figure 11).

The insufflation pressure is then decreased to 5-mm Hg to confirm haemostasis. It is of great importance that one strives for meticulous control of lymphatics and excellent haemostasis to further reduce the risk of lymphocele and/or haematoma formation, which could potentially become infected. A closed suction drain is positioned in the most dependent (caudal) portion of the lymphadenectomy such that fluid tends to find the drain when the patient is upright. Trocar incisions are closed in standard fashion.

For the left-side procedure, the robot maintained the same position (patient’s left side) and the trocars position is the same that the right side but in mirror mode. The docking is done at this trocar’s position.

**Figure 12.**
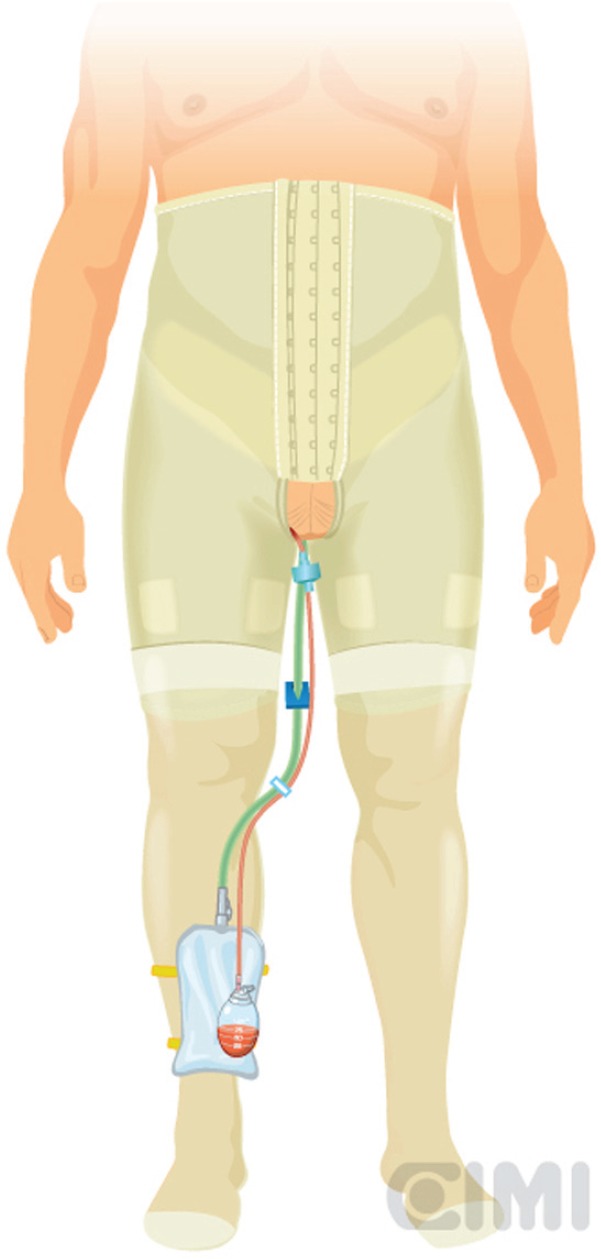
Placement of elastic compression stockings, showing drain output and Foley catheter.

The patient is allowed to ambulate the day of surgery and given a regular diet. Discharge is planned for the first postoperative day. A compressive elastic girdle, used for liposuction patients, is utilised to provide bilateral compression of the groins. In addition, elastic compression stockings were used since the first postoperative day until three months following surgery (Figure 12). Broad spectrum antibiotics are continued until after drains have been removed. Drains typically stay in place until output is <50/ml per 24-h period. All the patients receive DVT/PE prophylaxis typically using heparin or low molecular weight heparin for 15 days.

## Results

The entire procedure was performed with the robot-assisted technique. The length of operative time was 360 min; console time was 90 min for left lymphadenectomy and 150 min for the right side.

The median estimated blood loss was 100 ml, 50 ml in the right side and 150 ml in the left side. The hospital stay was three days.

The patient presented a left-side lymphocele that was drained during follow-up; drainage was removal at 21 days after the drainage was <50 cc/day.

The histopathological study reported squamous carcinoma metastases in one of 19 lymph nodes of the right side and two of 14 lymph nodes on the left side.

Considering these pathology results, we performed a laparoscopic pelvic lymphadenectomy reporting 11 negative nodes.

## Discussion

Traditional open inguinal lymphadenectomy is associated with significant potential morbidity; the most significant complications include lymphocele, wound infection, and skin flap necrosis. Patients undergoing prophylactic classical inguinal lymphadenectomy are considered to have an overall complication risk approaching 50% (range: 24–87%), including historical mortality of 1–3% [2].

In a recent series of inguinal lymphadenectomy, the overall complication rate was 49%, approximately evenly split between minor and major complications (48% minor and 52% major) [3]. Attempts to reduce the morbidity and mortality of lymphadenectomy include better patient selection, or using nomograms to inform decision making [4]. Other avenues include improving the technique by decreasing the area of dissection without compromising the oncological principles, sentinel node determination, and finally, the use of endoscopic lymphadenectomy [5].

Mathevet *et al* presented an interesting experience with a gasless endoscopic approach for inguinal dissection in vulvar and distal vaginal carcinoma. This is probably the most extensive experience for the inguinal endoscopic approach, and they reported low morbidity with a single intraoperative vascular injury. Perioperative complications were 25%, which included seven patients with lymphoceles [5].

In urology, in 2003, Bishoff *et al* [6] were first reported the use endoscopic inguinal node dissection, in two cadavers and one patient. Conversion occurred in this one patient because of inability to adequately mobilise the nodal mass superiorly. Further endoscopic approaches were described in 2006 by Tobias-Machado *et al* [7] who reported ten patients undergoing bilateral lymphadenectomy for nonpalpable lymph nodes. Standard open lymphadenectomy was performed on one side and endoscopic on the other. Nodal counts were similar, with 20% of complications on the endoscopic side, compared with 70% with open surgery, p 0.011. Skin-related complications were reduced from 50% to 5% with the endoscopic approach 5% [8]. Sotelo *et al* [9] reported the outcomes after 14 inguinal endoscopic lymphadenectomies in eight patients with clinical Stage T2 squamous cell carcinoma of the penis, with a median operative time of 91 min and an average node yield of nine. No wound-related groin complications occurred.

Master *et al* [10] described 25 endoscopic groin dissections. The mean number of lymph nodes retrived was nine. Complications occurred in 12% (a groin seroma requiring further drainage in one patient and cellulitis in two patients). Subsequently, the same group reported a detailed analysis of immediate and long-term complications associated with the procedure using the Clavien classification system. A total of 29 patients underwent endoscopic inguinal lymphadenectomy for cutaneous malignancies of the genitalia and lower extremities. Minor complications included mild to moderate leg oedema, seroma formation not requiring intervention, minimal skin edge necrosis requiring no therapy, and cellulitis managed with antibiotics. Major complications included death, sepsis, venous thromboembolism, re-exploration or other invasive procedures, severe leg oedema interfering with ambulation, skin flap necrosis, and rehospitalisation. A total of 41 endoscopic groin dissections (12-single session bilateral) were performed in 29 patients with a median follow-up of 604 days. There were no perioperative deaths. A total of 11 (27%) minor and six (14.6%) major complications occurred [11].

Recently, a group in India has reported similar experiences [12]; in ten patients, no skin-related complications were seen. Lymphocele was seen in 2/10 (20%) of patients, lymph node yield was 7–12 lymph nodes. A Chinese group [13] performed 11 lymphadenectomies, with two cases of minor complications; one patient had a seroma requiring needle aspiration and one lymphocele. Lymph node yield averaged 12.3 (range: 7–15) nodes per leg, among which mean deep nodes of 1.1 (range: 0–3) were included [13].

In 2009, a case report of the first staged bilateral endoscopic operation was performed robotically by Josephson *et al* [14]. Pathological examination revealed no metastatic involvement in six superficial and four deep lymph nodes. The contralateral dissection occurred weeks later, and pathological examination revealed no metastatic involvement in five superficial and four deep lymph nodes. The patient had no wound or lower extremity lymphoedema. Dogra *et al* [15] then described two dissections for palpable adenopathy, ipsilateral superficial, and deep dissections, followed by the contralateral side two days later. No postoperative complications developed and nodal yield was not reported.

Matin *et al* [16] performed a thorough evaluation of the adequacy of a robotic node dissection by subsequently opening the incision and having a separate oncologist look for unretrieved residual nodal tissue. They described ten such cases. The verifying surgeon’s role was to inspect the surgical field and, with additional dissection if necessary, ensure that no additional superficial inguinal lymph nodes (e.g., above the fascia lata of the thigh) remained within the operative field. If additional tissue was removed, it was sent to the pathology laboratory and assessed to define whether it was nodal in origin and whether it contained metastasis. In one of these inguinal fields, two residual lymph nodes were recovered from below Scarpa’s fascia along the superficial aspect of the inguinal field near the spermatic cord. No metastases were detected in these additional nodes. Among all the patients undergoing robotic dissection, 18 of 19 fields (94.7%) were adequately dissected.

In summary, the evidence from small series [6–10], comprising a total of 41 endoscopic groin dissections, [11] suggests that the morbidity of an endoscopic dissection is lower than open contemporary series [3]. A similar number of nodes can be retrieved [7], and more importantly, when an independent surgeon ‘audited’ the dissection, the field was adequately dissected in 94.7% [18]. The applicability of the robot is newer in this field so only a small series have been presented [14–18], and will need prospective evaluation in comparison with standard laparoscopic endoscopic procedures.

## Conclusion

In conclusion, the worldwide experience of endoscopic laparoscopic or robotic bilateral inguinal lymphadenectomy for penile cancer is still in its infancy. Early reports suggest that it is feasible, safe, and affords an oncologically appropriate dissection. Initial results are promising, but the dissection is best reserved for experienced minimally invasive surgeons.

Prospective studies with a standardised technique are required with larger cohorts and long-term follow-up to assess the results of the robotic procedure in comparison with open and laparoscopic techniques. Theoretically, with smaller incisions if the correct space is dissected, challenging skin flap complications that can occur with open surgery may be avoided. Robotic dexterity is useful when dissecting in this confined space. Oncological principles appear to be maintained.
